# Supervisors' Strategies to Facilitate Work Functioning among Employees with Musculoskeletal Complaints: A Focus Group Study

**DOI:** 10.1155/2015/865628

**Published:** 2015-08-25

**Authors:** Tove Ask, Liv Heide Magnussen

**Affiliations:** ^1^Department of Global Public Health and Primary Care, Physiotherapy Research Group, University of Bergen, P.O. Box 7800, 5020 Bergen, Norway; ^2^Department of Occupational Therapy, Physiotherapy and Radiography, Faculty of Health and Social Sciences, Bergen University College, P.O. Box 7030, 5020 Bergen, Norway

## Abstract

*Aim*. To explore what strategies the supervisors found beneficial to prevent or reduce sickness absence among employees with musculoskeletal complaints. *Methods*. Five focus groups were conducted and 26 supervisors from health and social sector participated. Commonly used strategies to prevent sickness absence and interdisciplinary cooperation in this work were discussed in the focus groups. Systematic text condensation was used to analyse the data. *Results*. The supervisors described five strategies for sick leave management: (1) promoting well-being and a healthy working environment, (2) providing early support and adjustments, (3) making employees more responsible, (4) using confrontational strategies in relation to employees on long-term sick leave, and (5) cooperation with general practitioners (GPs). *Conclusions*. Strategies of promoting a healthy working environment and facilitating early return to work were utilised in the follow-up of employees with musculoskeletal complaints. Supportive strategies were found most useful especially in the early phases, while finding a balance between being supportive, on one side, and confronting the employee, on the other, was endeavoured in cases of recurrent or long-term sick leave. Further, the supervisors requested a closer cooperation with the GPs, which they believed would facilitate return to work.

## 1. Introduction

Environmental and organizational factors in the workplace have been highlighted as important in the prevention of long-term sickness absence [1–5], where musculoskeletal complaints are the most frequent reasons for sick leave [6, 7]. Supervisors' responsibility and role have been emphasised in this work [8–13]. They are often the first to notice employees' health problems in the workplace and have an opportunity to make adjustments at an early stage in order to limit work disabilities [3, 13].

Promotion of employees' health and well-being has been linked to increased work ability and work participation [14, 15]. The impact of social support from supervisors has been emphasised in particular [15, 16]. Social support includes general social support at work, good communication and social contact with supervisors, a good work atmosphere, understanding of pain, help when things are difficult, and social support away from work [15].

Supervisor support may also influence the return to work (RTW) process. Poor supervisor support combined with high psychosocial demands has been found to be strongly associated with increased sickness absence due to overstrain or fatigue [16] and with increased risk of musculoskeletal complaints [15]. Labriola et al. [17] showed that low supervisor support, measured at the workplace level, was associated with increased risk of long-term sickness absence. However, other authors have reached opposite conclusions, demonstrating that low supervisor support was associated with a higher RTW rate [18], or have found no association between the level of supervisor support and risk of back pain and/or sick leave [19, 20].

Several aspects may influence the supervisors' choice of strategies in the follow-up of employees with health complaints. Tjulin and coauthors [21] found that workplace strategies shifted during three RTW phases: the prereturn, the initial return, and the postreturn phases. Supervisors seemed to follow the advice from the organisational policy in the RTW process, but when the employee was back in work they took less responsibility. Although assisting people with health complaints to stay at work has been recommended in the literature [2, 22], few have described this phase.

The choice of strategies may also be dependent on national legislation and policy. In Norway, both employer and employees have since 2011 been given increased responsibility in the RTW process. For instance, an early and close follow-up of employees on sick leave is considered important. After four weeks of sickness absence, the employer is responsible for facilitating work modifications and provides a detailed RTW plan in cooperation with the employee on sick leave. At seven weeks of sick leave, all stakeholders are required to participate in a dialog meeting in order to solve the problem [23].

Despite increased focus on the workplace and the supervisor's role in the prevention of sickness absence, knowledge is still lacking about key strategies utilised in the different phases of sick leave management, including the phase where the employee remains in work despite complaints. Insight into these strategies may increase our understanding of aspects that facilitate work participation. The aim was therefore to explore what strategies the supervisors used in the follow-up of employees with musculoskeletal complaints and what strategies they found most beneficial in the different phases of sick leave management.

## 2. Methods

The present study was part of the project “Function, Activity, and Work,” a joint project between the University of Bergen and the municipality of Bergen's Department of Health and Social Services. Focus group interviews were used since we wished to gain insight into the supervisors' experiences of following up employees with musculoskeletal complaints. Group discussions can stimulate the interaction among participants in the target group and yield a wide range of views across several groups [24].

The study was approved by the Regional Committee for Medical Ethics.

### 2.1. Participants

The participants were recruited from the Department of Health and Social Services in the municipality of Bergen, Norway. The department has around 7,000 employees, with a mean sickness absence rate of approximately 10% in recent years [25], which is considerably higher than the mean rate in Norway, which is 5.2% [7]. A total of 26 supervisors (23 women, three men), aged 31 to 62, who had worked as supervisors for from nine months to 18 years, agreed to participate. They were the immediate supervisors and, in addition to overall professional responsibility in their department, had responsibility for following up employees on sick leave. Most of them were nurses and a few were social educators. They worked in the home nursing service, nursing homes, or group homes for intellectually disabled people. Each supervisor had responsibility for about 40 employees.

### 2.2. Procedure

Eligible supervisors were given verbal and written information about the project and were invited to participate through their manager and the project managers. The supervisors contacted the project managers if they agreed to participate. Written informed consent was obtained from all participants before the start of the study.

Five focus groups were conducted between January 2012 and February 2013, with six to seven participants in each group. Three focus groups were carried out at the beginning of 2012, with two additional groups a year later. To get an impression of the strategies used over time, the supervisors in the first focus groups were invited to participate one year later, and six of them agreed. An additional seven supervisors were therefore recruited and mixed with the previous participants in two groups.

The focus groups took place in a conference room at the university and lasted for 90 to 110 minutes. All focus group discussions were led by a moderator (TA), and a comoderator (LHM) took field notes, describing the atmosphere and the interaction in the group discussions.

A semistructured interview guide with open-ended questions was used. The interview guide covered questions about strategies used in the follow-up of employees with musculoskeletal complaints and experiences of interdisciplinary cooperation in this work. The moderator guided the focus group discussions and encouraged all group members to participate. The comoderator summarised the main topics that emerged, and the participants were asked to elaborate on and/or confirm them.

### 2.3. Data Analysis

The data was analysed using systematic text condensation as described by Malterud [26] in four steps: (i) listening to the interviews and reading all the materials to get an overall impression and describe themes; (ii) identifying units of meaning relating to experiences and strategies when following up employees with musculoskeletal pain and coding them; (iii) systematic abstraction of meaning units by condensing the contents of each code group; and (iv) synthesising from condensation to generalised descriptions and concepts describing supervisors' experiences.

Both authors discussed the themes and their interpretations of the interview data. They met several times to discuss the transcripts and the open codes that were identified by the individual researchers until consensus was reached about the different codes. To validate data and to ensure that important aspects were not lost, all the transcripts were reread.

## 3. Results

The supervisors described different strategies related to three phases in the sick leave management and five corresponding themes: phase (1), preventive strategies for all employees: promoting well-being and a healthy environment; phase (2), supporting employees with musculoskeletal complaints to remain in work: providing early support and adjustments; and phase (3), RTW phase: making employees more responsible, using confrontational versus supportive strategies in relation to employees on long-term sick leave, and cooperation with the general practitioners (GPs).

*Phase 1*

*Promoting Well-Being and a Healthy Working Environment.* The most basic strategy to prevent sick leave was to ensure a well-functioning social climate in the workplace, which was considered to promote health and well-being. One strategy mentioned was to pay attention to each individual in their day-to-day work, while another was to strive for a good relationship with the employees. A safe and open atmosphere based on mutual trust and respect made it easier for employees to support each other, for example, if a colleague experienced musculoskeletal pain. One of the supervisors said:

*We have a positive working climate and know each other well. The working relationship is good and we try to help each other in our day-to-day work. *

Having a positive attitude to the workplace was also considered important in a well-functioning working climate. Among other things, this entailed being a role model, for instance, by taking part in the day-to-day work tasks together with employees, if necessary. Sometimes the supervisors organised workshops on the organisation's visions and goals in order to increase the feeling of belonging in the workplace. Educational courses were also regarded as a valuable way of inspiring and motivating employees to identify more with their work. A 35-year-old supervisor explained:

*You have to be aware, place people where they have their competence and interests… so that they get their inspiration back, - then a positive spiral will start. If they are motivated, it will be easier to continue working even if they have some pain.*

*Phase 2*

*Providing Early Support and Adjustments.* The supervisors agreed that sickness absence could be avoided if they recognised early signals of health complaints and offered support and modified work. The supervisors observed the employees in the performance of work tasks and gave advice on better working techniques. The sooner the supervisors became aware of employees' complaints the better, they explained. One of the supervisors put it as follows:

*I pay a lot of attention to their (employees') body language…and I try to follow them closely and, for instance, ask about complaints if somebody holds their hands to their back. I consider myself observant and pick up on many things. For me, this is one important way of prevention.*

Much time was spent on finding solutions and adjusting tasks to individual needs in order to prevent sickness absence. Easier work tasks, extra aids, working in pairs with the heaviest patients, changing shifts for a period, or, as a last resort, finding an alternative workplace in the municipality was among the solutions offered. Cooperation with the occupational health service was seen as helpful in many of these cases.

*She (the employee) told me that she had to be on 50% sick leave because she needed to be treated by a physiotherapist. I asked her if there was anything we (the workplace) could do to prevent the absence. Looking at the job plan together, we changed her shifts for a period. She was also helped by the occupational health service and avoided sickness absence.*

Although the supervisors showed great commitment, they expressed frustration about spending so much time and effort dealing with sick leave matters. The supervisors questioned the extent of their responsibility to adjust working conditions, especially in cases where the employees' main problem did not appear to be work-related. 

*Phase 3*

*Making Employees More Responsible.* Several supervisors argued that employees need to take more responsibility for themselves, both in the workplace and in life situations in general. A healthy lifestyle was encouraged. Being aware of the balance between work and private life and keeping in good physical shape were seen as a prerequisite for this challenging work. A 50-year-old supervisor had told one of her subordinates:

*If we intend to work as nurses until retirement, we'll have to do a lot of things like exercising and organising our private lives in a better way. *

Employees on sick leave were encouraged to be more responsible in their own RTW process, for instance, by phoning when they received a sick note, participating in meetings, and cooperating with the supervisor to find solutions that could facilitate an early RTW. Statutory requirements, such as preparing a detailed RTW plan and holding a dialogue meeting after a certain time, were seen as useful in this process. When clarifying the responsibility each part had and the consequences of not following the procedures, the supervisors found support in the written rules at meetings with their subordinates. One of the younger supervisors stated:

*I think it is okay to make demands of employees even if they are on sick leave. We can be better at that…It can easily happen that we do not dare to ask critical questions. We have to emphasize not only the rights, but also the duties of employees on sick leave in connection with sickness absence.*

*Confrontational versus Supportive Strategies in relation to Employees on Long-Term Sick Leave.* The supervisors agreed that a supportive attitude would facilitate a healthy working environment and thereby prevent sickness absence. However, they disagreed on the most appropriate strategy for following up employees on recurrent or long-term sick leave, and these differences were eagerly discussed in the groups. While some were convinced that being understanding and supportive was the best approach to helping the employees to return to work, others believed that they as supervisors could be too supportive and claimed that, in some cases, a confrontational style was more useful.

Confrontational discussions were especially useful in communication with some of the younger employees and employees with recurrent periods of sick leave who they believed exploited the sickness certification system. In such cases, they were more direct towards the employees and asked critical questions about their sickness absence. They sometimes increased the pressure on the employees by informing them about and discussing financial consequences of being on long-term sick leave and difficulties finding jobs in future. They expressed a lack of trust in those who returned to work after one year's absence when further sick leave would have resulted in reduced disability payments. The supervisors suspected that these employees could manage to work well and wondered why they had not returned to work earlier. One supervisor claimed that, for temporary employees with a sick leave history, sickness absence decreased after these meetings:

*I had to explain to each of them that their high level of sickness absence was not in line with national guidelines for that illness and that frequent sickness absence would have consequences for future work.*

Some of the supervisors felt guilty, however, about questioning the employees' work ethic. The usefulness of confronting the employees nevertheless appeared to overshadow this feeling. Although the strategy of making demands and applying pressure was emphasised in some situations, supportive strategies were also valued, but the supervisors found it challenging to strike the optimal balance between the two.

*Cooperation with GPs.* Cooperation with GPs in dealing with challenging cases of sickness absence was seen as very important. The GPs' assessment of what the employees could safely do helped to reassure all parties in the dialogue. This led to a better evaluation of the employee's work ability and provided support when planning modified work tasks. Some supervisors said that these meetings had often resulted in an earlier return to work or at least from full-time to part-time sick leave. The supervisors found, however, that GPs knew little about current opportunities for adjusting work tasks and wanted closer dialogue with GPs in difficult cases. One of the younger supervisors said:

*In our jobs, we are working with multi-handicapped people and we have a lot of heavy lifting, but it is not black or white. It is often possible to find easier tasks for a while, but the doctors know too little about our workplace, and people listen to what the doctors recommend. *

As a consequence, employees risked being put on sick leave without having tried other alternatives first.

## 4. Discussion

In the present study, we explored what strategies the supervisors found beneficial to prevent sick leave among employees with musculoskeletal complaints. All supervisors found supportive strategies useful to promote health and well-being and early RTW. For employees on recurrent or long-term sick leave, some supervisors found these strategies useful as well, while others emphasised a more confrontational style. Striking a balance between being supportive, on the one hand, and making demands and confronting the employees, on the other hand, seemed to be challenging.

The supervisors expressed great enthusiasm and involvement in the follow-up of employees with musculoskeletal complaints. Although most attention was directed towards employees who had already developed musculoskeletal complaints, strategies for creating a good working environment for all employees were also eagerly discussed.

The strategies the supervisors claimed to apply seemed to follow a specific pattern that was related to the different phases in the sick leave management. Mainly supportive strategies were used to promote a healthy working environment and to help employees remain in work despite musculoskeletal complaints. These strategies included facilitating a good social climate at work, taking notice of each individual, assisting in work situations, making modifications, and offering educational courses. They also motivated the employees to engage with the organisation's visions and strategy plans, which was considered to increase well-being and positive attitudes towards the workplace. Our findings are in line with other studies that point to the importance of social support from supervisors to enable employees to remain in work. An in-depth review of 52 studies showed that social support from coworkers and/or supervisors could help employees to cope with their musculoskeletal complaints and thus have impact on the prevention of musculoskeletal complaints and sickness absence [15]. According to a Dutch study [9], workers and occupational physicians as well as supervisors considered the supervisor's role to be important in relation to optimising functioning at work and helping workers with health problems to stay at work. In two systematic reviews, it was found that supervisor support did not seem to prevent the development of back pain [19, 20]. In our study, however, the focus was on management of musculoskeletal complaints and prevention of sick leave due to complaints, not on primary prevention.

There is conflicting evidence as regards which strategies are most appropriate in the RTW phase. Supportive strategies have been considered to be beneficial in the RTW process [2, 3, 10, 13, 27]. Employees on sick leave who reported that leadership qualities were good returned to work sooner than those not reporting such qualities among their leaders [27]. The feeling of being protected by their supervisor was the strongest predictor of early RTW. However, other studies have shown no association between supervisor support at work and sickness absence due to back pain [19, 20]. Post et al. [18] also found that low supervisor support was associated with a higher RTW rate. One explanation may be that having a supportive and empathetic supervisor could make it easier for employees to extend their sick leave and also lead to a feeling of dependency on the supervisor [18].

In the present study, utilising supportive strategies alone was not regarded to be sufficient to reduce sick leave. Confronting employees and demanding more responsibility of them in the RTW process were considered to be essential by some supervisors. Making demands on employees on sick leave was also emphasised by the supervisors in a Swedish study [10]. However, encouragement and pressure to remain in or return to work may have negative consequences for health and well-being if the employees are too ill to work [28]. The supervisors in our study seemed to be aware of this, and close cooperation with the GPs was seen as desirable in order to get a better judgment of the employees' work ability.

In line with our findings, previous research has concluded that close collaboration between health professionals, employees, and supervisors is essential in the RTW process [1–3, 22]. From supervisors' point of view, cooperation with health professionals was considered to be helpful in selecting modified work tasks, establishing a mutual understanding, and clarifying the supervisor's role in the RTW process [1, 10, 11]. However, the supervisors in the present study claimed that GPs knew too little about the workplace and they wanted a closer dialogue with the GPs in order to increase the effectiveness of the RTW efforts. According to a systematic review, employers described that the physicians were difficult to reach when they needed to discuss the employee's capability in relation to work tasks [3]. It is argued that GPs could make better judgments about work ability if they collaborated with the employer to obtain information about the individual's work situation [29].

Supervisors may find it challenging to strike a balance between production demands and employees' health concerns [1, 10, 12], and this could possibly influence their strategies in the RTW process. Swedish workers and supervisors were interviewed about the employers' role in relation to RTW, and both groups reported that the economic consideration for their company often dominated at the expense of the legal and ethical aspects [12]. This may also be the reason why some supervisors in the present study preferred to use confronting strategies in difficult RTW cases.

The supervisors in our study expressed great enthusiasm about following up employees with musculoskeletal complaints. Their preoccupation with sick leave and disability prevention is in accordance with the Norwegian government's policy and with legislation on the follow-up of employees on sick leave [23]. The high level of sickness absence in this department might therefore seem surprising. However, Health and Social Services is the sector with the highest sickness absence rate in Norway [7]. Nursing homes and home nursing services are characterised by female employees, physically demanding work, low control, and night shifts, all described as risk factors for prolonged sickness absence [30–32]. It is therefore likely that the high sick leave level, in this particular department, reflects the level in such workplaces in Norway in general.

### 4.1. Methodological Considerations

Five group interviews were conducted with six to seven participants in each group in accordance with common recommendation for optimal group interaction [33]. The data yielded rich and broad descriptions that shed light on the topic, and we considered the material to have reached saturation since no new insights emerged from the last interviews.

We chose a systematic text condensation of the qualitative interview material and carried out an analysis of meaning across the interviews [26]. Two focus groups interviews were conducted one year after the first ones. This gave us more nuanced and broad descriptions, but no new strategies appeared. Because we noticed no time effect in this context, we considered transversal analysis to be the best method.

Half of the participants were invited to participate by their managers and half were self-selected. This may have resulted in selection sample of participants who were more enthusiastic and motivated than others in relation to finding solutions for the employees with musculoskeletal complaints.

The study was limited to the Bergen area and the supervisors were healthcare workers, mainly female nurses. Including male employees and employees from other professions might have provided additional perspectives. Substantial differences in culture, social insurance system, and sickness certification legislations between different countries may also influence the choice of strategies. However, how supervisors follow up employees with health complaints is a general topic, and knowledge from this study may be useful to others involved in prevention of sickness absence.

## 5. Conclusion

The present study provides insight into strategies used by supervisors to facilitate work functioning among employees with musculoskeletal complaints. Different strategies were applied depending on the phase of sick leave management. Supportive strategies were found most useful especially in the early phases, while finding a balance between being supportive, on one side, and making demands and confronting the employees, on the other side, was endeavoured in cases of recurrent or long-term sick leave. Furthermore, the supervisors requested a closer cooperation with the GPs, which they believed would facilitate RTW.
